# Seropositivity of Hepatitis B virus and Hepatitis C virus dual Infection among blood donors in Nyala Teaching Hospital

**DOI:** 10.1186/1743-422X-6-227

**Published:** 2009-12-22

**Authors:** Mustafa Abdalla Ali Abou, Yassir Mohammed Eltahir

**Affiliations:** 1 Department of Laboratories Administration, State Ministry of Health, South Dar Fur, Nyala, Sudan; 2 UNAMID Level 1 Clinic, Nyala, South Dar Fur, Sudan; 3 Department of Preventive Medicine & Veterinary Public Health, Faculty of Veterinary Science, University of Nyala, Nyala, Sudan

## Abstract

The aim of this study was to determine the seropositivity of Hepatitis B virus (HBV) and Hepatitis C virus (HCV) dual infection among blood donors in Nyala Teaching Hospital, which is the biggest (400 beds) hospital in great Dar Fur of Western Sudan. 400 blood donors were tested serologically for the detection of HBsAg and anti-HCV antibodies. Only one (0.25%) out of the 400 examined blood donors was detected reactive for both HBsAg and anti-HCV antibodies. The study concluded that the seropositivity of HBV and HCV dual infection among population studied is uncommon.

## Introduction

Dual infection with HBV and HCV leads to more aggressive liver disease [1]. In addition to chronic liver disease, coinfection of HBV and HCV is frequently found in injection drug users (IDU, 42.5%) [2], patients on hemodialysis (3.7%) [3], patients undergoing organ transplantation (8%) [4], HIV-positive individuals (66%) [5], and beta-thalassemia patients (10%) [6], which means that those are the high risk population for infection of HBV and HCV concurrently. As HBV and HCV have the same transmission routes, dual infection may occur [7]. No published data of the seropostivity of HBV and HCV dual infection in great Dar Fur, and may be in the large Sudan. So the current study amied to determine the seropositivity of HBV and HCV dual infection among blood donors in Nyala Teaching Hospital.

## Materials and methods

This study was conducted during the period from May to July 2007, in Nyala Teaching Hospital, which is the biggest (400 beds) hospital in great Dar Fur, and according to the blood bank records 3600 pints of blood are collected annually from blood donors. 400 male, apparently healthy blood donors were randomly selected and enrolled in this study. Five ml of blood were drawn from each subject; sera were separated, aliquoted, labeled within two hours of collection and stored at -70°C. Serum samples initially tested for HBsAg and anti-HCV antibodies with Immunochromatographic Test (ICT) from Advanced Quality, then screened with a 3^rd ^generation Enzyme Linked Immunosorbent Assay (ELISA), Equi-HBsAg and EIAgen anti-HCV antibodies from Equibar and Adalits respectively.

## Results

A total 400 male blood donors were enrolled in this study, with a mean age of 33 years and an age range of 18-49 years. The seropositivity of HBV and HCV dual infection was detected in only one (0.25%) blood donor.

## Discussion

Surveillance of carriers of viral hepatitis is essential to assess the burden of the disease in the population. Although dual infection with HBV and HCV is not uncommon in the geographic areas where a high endemic level of both infections is reported, such as Southeast Asia and the Mediterranean, the role of this dual infection in the pathogenesis of chronic liver disease is still controversial (8,9,10,11). Despite dual infection with HBV and HCV leads to mutual suppression of both viruses, several studies have suggested that HBV and HCV infection may be associated with a more severe clinical presentation [12,13]. The findings of this study showed that the seropositivity of dual infection of HBV and HCV among blood donors in Nyala Teaching Hospital was (0.25%), this percent is in accordance with the endemic level of both viruses in South Dar Fur State, Sudan, in which the seroprevalence of HBV is of an intermediate level (6.25%) and HCV seroprevalence of low level (0.65%)[14]. Dual infection of HBV and HCV in Nyala when compared with studies conducted in other parts of Sudan, there is afew published data indicating that dual infection of HBV and HCV was never detected in Northern Sudan[15]. So dual infection of HBV and HCV is uncommon in Nyala and may be in the large Sudan due to the endemic level of both viruses.

## Conclusion

The study concluded that the seropositivity of HBV and HCV dual infection among population studied is uncommon.

## Competing interests

The authors declare that they have no competing interests.

## Authors' contributions

MAAA carried out the whole work of the study. YME supervised the work of the study. All authors read and approved the final manuscript.
